# The role of c-Myc-RBM38 loop in the growth suppression in breast cancer

**DOI:** 10.1186/s13046-017-0521-5

**Published:** 2017-04-11

**Authors:** Xiao-Xia Li, Liang Shi, Xu-Jie Zhou, Jing Wu, Tian-Song Xia, Wen-Bin Zhou, Xi Sun, Lei Zhu, Ji-Fu Wei, Qiang Ding

**Affiliations:** 1 Jiangsu Breast Disease Center, the First Affiliated Hospital with Nanjing Medical University, 300 Guangzhou Road, Nanjing, 210029 China; 2 Research Division of Clinical Pharmacology, the First Affiliated Hospital with Nanjing Medical University, 300 Guangzhou Road, Nanjing, 210029 China

**Keywords:** Breast cancer, c-Myc, RBM38, mRNA stability, Growth suppression

## Abstract

**Background:**

RNA-binding protein 38 (RBM38) is a member of the RNA recognition motif (RRM) family of RNA-binding proteins (RBPs). RBM38 often exerts its function by forming regulatory loops with relevant genes. c-Myc is an oncogenic transcription factor that is upregulated in one-third of breast cancers and involved in many cellular processes in this malignancy. In our previous study, RBM38 was identified as a tumor suppressor in breast cancer. In the present study, we investigated the mechanisms underlying the regulation of this tumor suppressor.

**Methods:**

Lentivirus transfections, Western blotting analysis, qRT-PCR and immunohistochemistry were employed to study the expression of c-Myc and RBM38. Chromatin immunoprecipitation and dual-luciferase reporter assays were performed to investigate the direct relationship between c-Myc protein and the RBM38 gene. RNA immunoprecipitation combined with dual-luciferase reporter assays was conducted to confirm the direct relationship between the RBM38 protein and the c-Myc transcript.

**Results:**

Knockdown of c-Myc increased RBM38 expression by binding directly to specific DNA sequences (5′-CACGTG-3′), known as the E-box motif, in the promoter region of RBM38 gene. Additionally, RBM38 destabilized the c-Myc transcript by directly targeting AU-rich elements (AREs) in the 3′-untranslated region (3′-UTR) of c-Myc mRNA to suppress c-Myc expression. Moreover, specific inhibitors of c-Myc transcriptional activity inhibited RBM38-induced suppression of growth, implying that RBM38 acts as a tumor suppressor via a mechanism that depends, at least partially, on the reduction of c-Myc expression in breast cancer.

**Conclusions:**

RBM38 and c-Myc form a unique mutually antagonistic RBM38-c-Myc loop in breast cancer.

**Electronic supplementary material:**

The online version of this article (doi:10.1186/s13046-017-0521-5) contains supplementary material, which is available to authorized users.

## Background

The incidence of breast cancer in women has increased rapidly in recent decades [1], ranking as the second most commonly diagnosed cancer in women aged 30–59. In China, breast cancer is the major cause of cancer deaths in women younger than 45 [2]. Breast cancer is a complex disease, associated with numerous genetic aberrations, including inactivation of various tumor suppressor genes and oncogene activation [3].

RNA-binding proteins (RBPs) mediate post-transcriptional regulation of target genes via a variety of mechanisms including stability, polyadenylation, transport, translation and RNA splicing, and also act as tumor suppressors or oncogenes in many cancers [4]. As a member of the RNA recognition motif (RRM) family of RBPs, RNA-binding protein 38 (RBM38, also called RNPC1) has been shown to function as a tumor suppressor in breast cancer [5], acute myeloid leukemia [6], colorectal cancer [7], and correlates with improved survival in human ovarian cancer [8]. RBM38 has been implicated in stabilization of p21, p73 and Hu antigen-R (HuR) transcripts and destabilization of mouse double minute 2 homolog (MDM2) transcripts, via binding to AU/U-rich elements (AREs) in the 3′-untranslated region (3′-UTR) of their mRNAs. These interactions result in the suppression of tumor growth [9–11]. Moreover, RBM38 exerts its function by forming regulatory loops with relevant genes, such as p53 [9, 12], p63 [9, 13], p73 loop [9, 11] and E2F1 [8]. For instance, RBM38, as a potential common target gene of members of the p53 family (p53, p63γ and p73β) [14], contains two p53-responsive elements (p53REs), p53RE-1 and p53RE-2 [9]. RBM38 regulates these p53 family members (p53, p63, p73) by binding directly to these specific regions in the 3′-UTRs of their mRNA [11–13] to form a regulatory loop.

In our previous study, ectopic expression of RBM38 could inhibit breast tumor cell proliferation, suppress tumor cell migration and invasion in breast cancer cells, acting as a tumor suppressor [5]. Moreover, RBM38 increases the expression of p21 and p73, resulting in cell growth arrest in the G1-phase in the MCF-7 breast cancer cell line [5, 9–11]. Few genes have been reported to participate in this process. c-Myc acts as an oncogenic transcription factor that promotes G1-phase cell cycle progression by increasing the activity of cyclin-dependent protein kinases 2, 4 and 6 [15–18]. The mechanism by which c-Myc exerts these effects depends on its binding to specific DNA sequences (5′-CACGTG-3′), known as E-box motifs, in the promoter regions of target genes [19–22]. c-Myc is upregulated in one-third of breast cancers [23], and is involved in various cellular process including cell growth, cell cycle control, metabolism, adhesion, differentiation and apoptosis [24]. Bioinformatics analysis revealed the presence of an E-box motif (sequence: 5′-CACGTG-3′) in the RBM38 gene, thus implicating RBM38 as a potential target gene of c-Myc. Moreover, c-Myc transcripts possess several AREs with RBM38-binding potential in the 3′-UTR of its mRNA. In addition, RBM38 has been identified as a tumor suppressor in breast cancer, and tends to form regulatory loops with its target genes. In this study, we investigated the hypothesis that RBM38 forms a regulatory loop with c-Myc to mediate the function of RBM38 in breast cancer and that this interaction occurs via a mechanism similar to that underlying the interaction of RBM38 with p53 family members.

## Methods

### Cell culture and transfection

Two human breast cancer cell lines, MCF-7 and ZR-75-1, were obtained from the American Type Culture Collection (ATCC, USA). The cells were grown in complete medium consisting of high glucose Dulbecco’s Modified Eagle Medium (DMEM, Wisent, China) supplemented with 10% fetal bovine serum (FBS), 100 μg/ml penicillin-streptomycin (Hyclone, USA). Cells were cultured at 37 °C in a humidified atmosphere containing 5% CO_2_ and 95% air. Lentivirus constructs for RBM38 overexpression and knockdown were obtained from GenePharma (China), and were generated as described previously [25].

### Western blotting analysis

Western blotting analysis was performed as described previously [26] using the following detection antibodies: anti-rabbit RBM38 (Santa Cruz, USA), anti-mouse c-Myc (Thermo, USA), anti-mouse β-actin (Cell Signaling technology, USA) and anti-rabbit and anti-mouse secondary antibodies (Cell Signaling technology, USA).

### RNA extraction, reverse transcription and quantitative RT-PCR (qRT-PCR)

RNA extraction, reverse transcription and qRT-PCR were performed as described previously [26] using primers listed in Additional file 1: Table S2.

### Chromatin immunoprecipitation (ChIP)

ChIP assays were performed using chromatin immunoprecipitation kits (17–371, EZ-ChIP, Millipore) according to the manufacturer’s instructions. Briefly, MCF-7 cells were plated in 15 mm culture dishes. The proteins were cross-linked with DNA by incubation with 37% formaldehyde (final concentration 1%) for 10 min at room temperature. Cross-linking was terminated by the addition of glycine solution (final concentration of 0.125 M). Subsequently, the cells were scraped from the culture dish, collected by centrifugation and resuspended in lysis buffer containing. Cell lysates were aliquoted (100 μl), and sonicated to shear DNA (average fragment size, 500 bp). After preserving 10 μl of the supernatants as Input material, the fractions were collected and incubated with protein G agarose for 1 h at 4 °C. Protein G agarose was pelleted and the fresh supernatants were reincubated with 5 μg primary antibody (anti-c-Myc (Thermo, USA) or normal mouse IgG) at 4 °C overnight with rotation. The supernatants were then reincubated with protein G agarose for 1 h at 4 °C. After washing the protein G agarose, two elution procedures were performed to obtain the protein-DNA complexes. Chromatin cross-linking was disrupted incubation at 65 °C overnight in the presence of 5 M NaCl. The immunoprecipitated DNAs and Input material were purified by treatment with RNase A and proteinase K. An aliquot (2 μl) of each sample was analyzed by PCR using specific primers (sense: 5′- AATATCGGTCGGATGAACTAAT-3′ and anti-sense: 5′-CGTCTGGTGAATTAAACGTAAA-3′).

### Dual-luciferase reporter assay

Dual-luciferase reporter assays were performed in triplicate using kits (Promega, USA) according to the manufacturer’s instructions. Briefly, 200 ng of a pGL3 reporter containing target regions and 5 ng of Renilla luciferase vector (pRL-TK; Promega, USA) as the internal control, were co-transfected into breast cancer cells. After 48 h, the cells were harvested to measure the luciferase activity.

### CCK-8 assay

Cell proliferation was evaluated using CCK-8 kits (Dojindo, Japan) following the manufacturer’s instruction. Briefly, 5 × 10^3^ cells suspended in 200 μl medium were seeded in triplicate in a 96-well plate. After incubation for 12 h, the cells were incubated for a further 72 h in the presence or absence of 70 nM c-Myc inhibitors (10058-F4 or 10074-G5). On the days of measuring the growth rate of cells, the medium in each well was replaced with 100 μl DMEM containing 10% CCK-8. The plates were incubated at 37 °C for 2.5 h and OD values at 450 nm were measured using a microplate reader (Groding, Tecan, Austria). Each test was performed in triplicate.

### Colony formation assay

Colony formation analysis was performed as previously described [5]. The breast cancer cells were seeded into 6-well plates (800 cells per well). After incubation for 24 h, the cells were incubated for a further 21 d in the absence or presence of 30 nM c-Myc inhibitors (10058-F4 or 10074-G5). The resulting colonies were fixed in 75% absolute ethanol for 60 s, washed twice with PBS and stained with Giemsa (Sigma, USA) for 15 min.

### RNA immunoprecipitation (RIP)

RIP was carried out as previously described [25]. RT-PCR and qRT-PCR were used to measure the levels of c-Myc and p21 transcripts in the RBM38 or IgG immunocomplexes.

### RNA electrophoretic mobility shift assay (REMSA)

To generate REMSA probes, three regions (A–C) in 3′-UTR of c-Myc mRNA and a region in 3′-UTR of p21 mRNA were amplified by PCR using primers containing the T7 promoter sequence (5′-TAATACGACTCACTATAGGG-3′). The sequences of the PCR products are listed in Additional file 1: Table S1. U to G mutations in relevant AREs were used as the mutant probes. The RNA probes were acquired by in vitro transcription with a MEGAshortscript kit (Ambion, USA) in the presence of biotin-16-UTP (Roche, Switzerland) according to the manufacturer’s instructions. REMSA was performed as previously described [26].

### RNA interference

Double-stranded siRNAs targeting c-Myc were obtained from GenePharma (China). MCF-7 and ZR-75-1 cells were transiently transfected with two c-Myc siRNA (designated siRNA1, siRNA2) and a control (designated CTRi) using Lipofectamine 3000 (GenePharma) according to the manufacturer’s instructions. Colony formation analysis was used to evaluate the effect of siRNA and Dual-luciferase reporter assays were performed at 48 h after siRNA treatment. The sequences of the siRNAs are as follows: siRNA1: sense 5′-UCCUGAGACAGAUCAGCAATT-3′ and anti-sense 5′-UUGCUGAUCUGUCUCAGGATT-3′; siRNA2: sense 5′-GGCGAACACACAACGUCUUTT-3′ and anti-sense 5′-AAGACGUUGUGUGUUCGCCTT −3′.

### Immunohistochemical (IHC) staining and analysis

Breast tissue samples were obtained from 162 patients who received treatment for breast cancer at the First Affiliated Hospital of Nanjing Medical University, China, between 2004 and 2007. The collection and use of the samples was reviewed and approved by the Institutional Ethics Committee of the First Affiliated Hospital of Nanjing Medical University. The TNM staging was defined according to the American Joint Committee on Cancer (AJCC) (6^th^ version, 2002). IHC staining of the same tissue samples with RBM38 and c-Myc antibodies was conducted and analyzed as previously described [26, 27].

### Immunofluorescence (IF)

The location of RBM38 and c-Myc was determined by the immunofluorescence (IF) analysis as previously described [25].

### Statistical analysis

Each experiment in this study was repeated in triplicate, unless otherwise specified. The data were analyzed using SPSS software (Version, 20.0). The correlation between RBM38 and the clinical pathological parameters was analyzed using *χ* ^2^ tests. The other data are presented as mean ± standard error of the mean (SEM) and differences between groups were analyzed by Student’s *t*-test. *P* < 0.05 was considered to indicate statistical significance.

## Results

### IHC staining of RBM38 and c-Myc in human breast cancer tissues

**Fig. 1:**
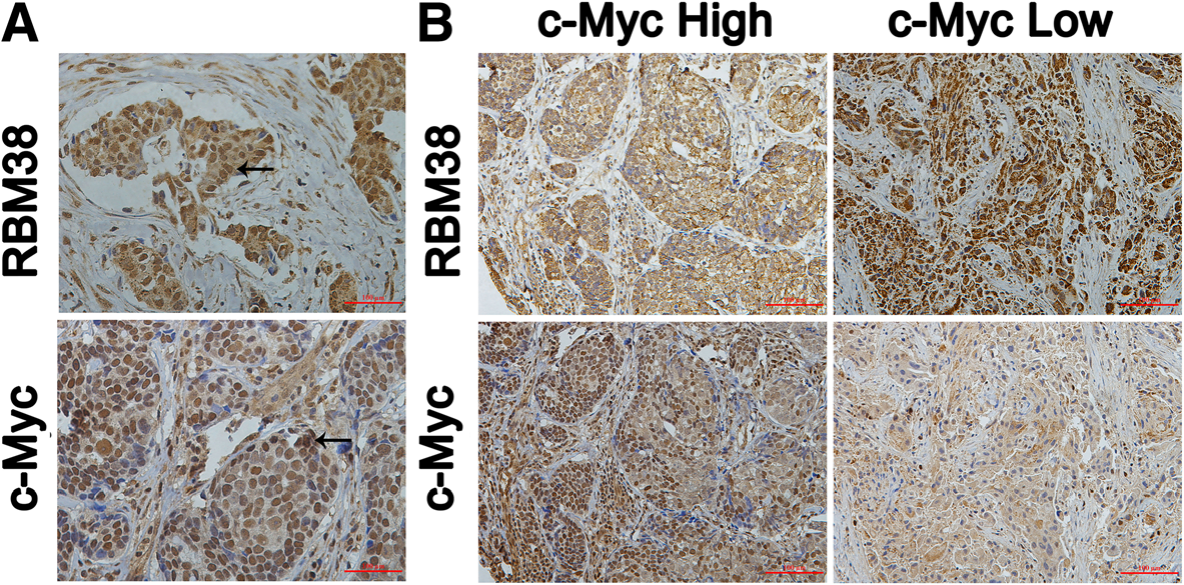
RBM38 expression correlates negatively with c-Myc in human breast cancer tissues. **a** IHC analysis of RBM38 and c-Myc expression in breast cancer at 400× magnification. RBM38 and c-Myc were expressed in the cytoplasm and nucleus (*black arrow*). *Scale bars* indicated 100 μm. **b** IHC analysis of RBM38 and c-Myc expression in breast cancer at 200× magnification. The breast cancer with the high staining of c-Myc expressed low level of RBM38; the breast cancer with the low staining of c-Myc expressed high level of RBM38. Scale bars indicated 100 μm

To explore the expression of the RBM38 and c-Myc, IHC staining was performed in 162 breast cancer tissues. RBM38 and c-Myc were expressed both in the cytoplasm and nucleus (Fig. 1a). Representative images of RBM38 expression in breast cancer tissues expressing high and low levels of c-Myc presented in Fig. 1b show that RBM38 expression was negatively correlated with c-Myc expression. The analysis of the correlation between RBM38 expression and clinicopathological features of the breast cancer patients is shown in Table 1. The cellular localization of RBM38 and c-Myc in the breast cancer cells was investigated using IF staining (Additional file 2: Figure S1).

**Table 1:** Association of RBM38 with c-Myc and clinicopathological characteristics of breast cancer

Clinicopathological characteristics	RBM38 expression			
	Cases (*n*)	Low (%)	High (%)	*P*-value
Age (years)				0.196
<50	66	31 (46.7)	35 (53.3)	
≥50	96	55 (57.3)	41 (42.7)	
Tumor size				0.629
≤2 cm	65	33 (50.8)	32 (49.2)	
˃2 cm	97	53 (54.6)	44 (45.4)	
TNM stage				0.127
I–II	128	64 (63.4)	64 (36.6)	
III	34	22 (87.5)	12 (12.5)	
c-Myc				0.014
Low	108	50 (46.3)	58 (53.7)	
High	54	36 (66.7)	18 (33.3)	

### c-Myc regulates RBM38 expression by binding to the E-box in the promoter region of RBM38 gene in breast cancer cells

**Fig. 2:**
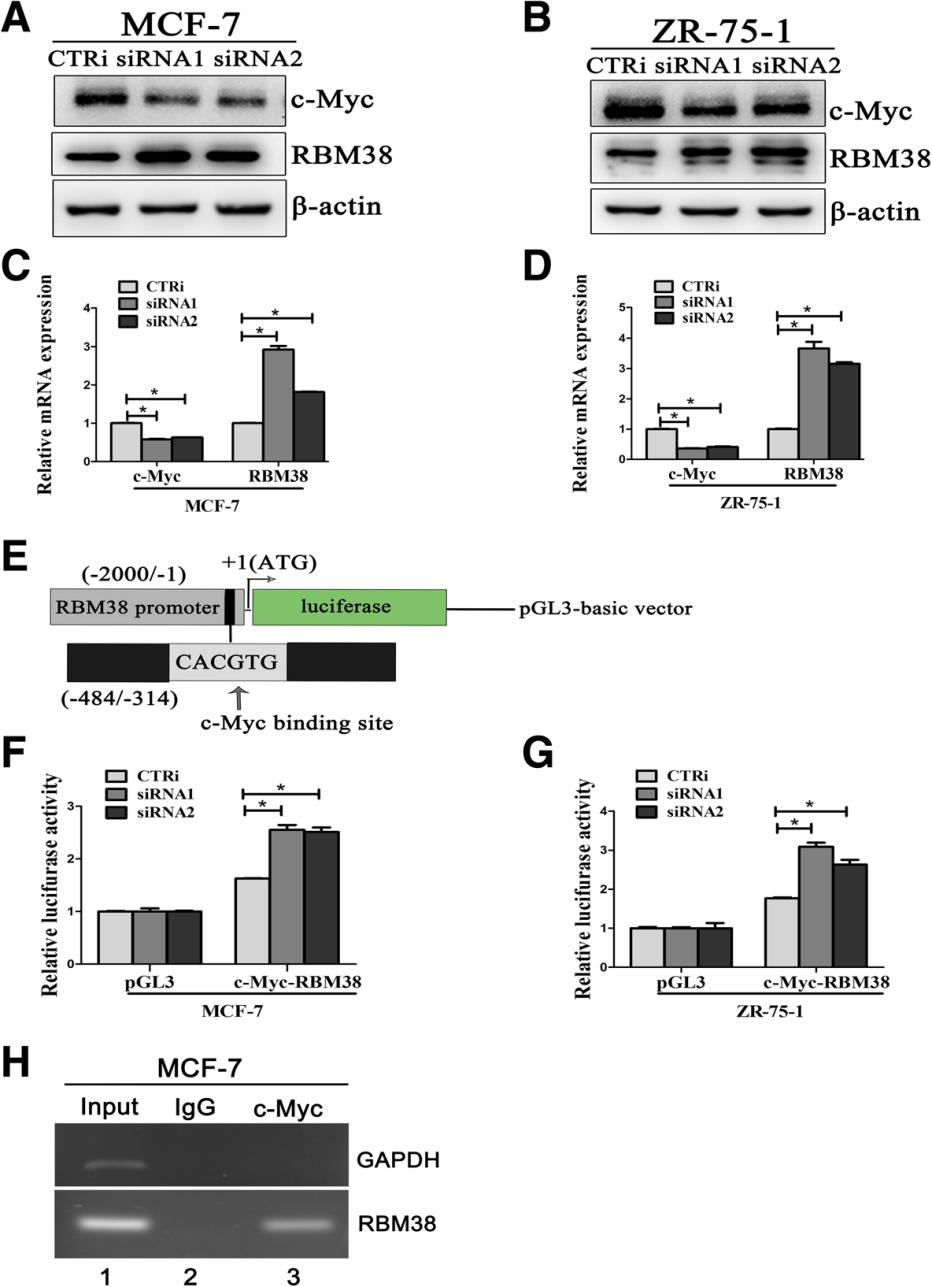
c-Myc regulates the RBM38 expression by binding to the promoter region of RBM38 gene in breast cancer cells. **a**–**d** MCF-7 and ZR-75-1 cells were transfected with two siRNAs to knockdown c-Myc (siRNA1, siRNA2) and the control (CTRi). RBM38 expression was obviously upregulated at both the protein (**a**, **b**) and mRNA levels (**c**, **d**) following c-Myc knockdown. **e** Schematic diagram of the luciferase reporter constructs containing the E-box in promoter region of RBM38 gene. **f** MCF-7 and **g** ZR-75-1 cells with c-Myc knockdown siRNA and the control were transfected with pGL3 control reporter or pGL3 reporter carrying the E-box in promotor region of RBM38 gene. The fold increased in relative luciferase activity was a product of the luciferase activity induced by c-Myc knockdown divided by that induced by the control. Results were representative of three independent experiments and presented as the mean ± SEM. *P < 0.05. **h** c-Myc could directly bind to E-box in promoter region of RBM38 gene. Lane 1, Input DNA; Lane 2, DNA from MCF-7 cells immunoprecipitated with normal mouse IgG; Lane 3, DNA from MCF-7 cells immunoprecipitated with anti-c-Myc antibody

RBM38 expression was obviously upregulated at both the protein and mRNA levels following c-Myc knockdown in MCF-7 (Fig. 2a, c; *P* < 0.05) and ZR-75-1 (Fig. 2b, d; *P* < 0.05) cells. Subsequent analysis of the RBM38 gene revealed the presence of an E-box motif (sequence: 5′-CACGTG-3′) with the potential to be bound specifically by c-Myc-Max complexes in its promoter region. ChIP assays showed that c-Myc bound to this E-box in MCF-7 cells (Fig. 2h). GAPDH, which represented a negative control, was not bound by c-Myc (Fig. 2h). Luciferase activity assays showed that luciferase activity for a reporter carrying the RBM38 E-box was prominently increased in the absence of c-Myc in MCF-7 (Fig. 2f) and ZR-75-1 (Fig. 2g) cells. This indicated that c-Myc negatively regulates RBM38 expression by binding to the E-box in the promoter region of the RBM38 gene in breast cancer cells.

### RBM38 regulates c-Myc expression in breast cancer cells

**Fig. 3:**
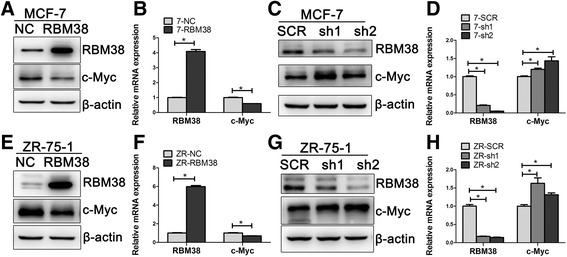
RBM38 represses c-Myc expression in breast cancer cells. **a**, **b**, **e**, **f** MCF-7 and ZR-75-1 cells were transfected with lentivirus to overexpress RBM38 (RBM38) and the control (NC). c-Myc expression significantly decreased at both the protein (**a**, **e**) and mRNA levels (**b**, **f**) following RBM38 overexpression. **c**, **d**, **g**, **h** MCF-7 and ZR-75-1 cells were transfected with lentivirus to knockdown RBM38 (sh1, sh2) and the control (SCR). c-Myc expression was significantly upregulated at both the protein (**c**, **g**) and mRNA levels (**d**, **h**) following RBM38 knockdown. Western blot and qRT-PCR were performed to detect the expression of RBM38 and c-Myc in protein and mRNA level. Results were representative of three independent experiments and presented as the mean ± SEM, *P < 0.05

To investigate the ability of RBM38 to regulate c-Myc expression in breast cancer, MCF-7 and ZR-75-1 cells were transfected with lentiviruses overexpressing either RBM38 (RBM38) or a control (NC). c-Myc expression significantly decreased at both the protein and mRNA levels following RBM38 overexpression in MCF-7 (Fig. 3a, b; *P* < 0.05) and ZR-75-1 (Fig. 3e, f; *P* < 0.05) cells. MCF-7 and ZR-75-1 cells were transfected with the control (SCR) or RBM38 knockdown (sh1, sh2) lentivirus. c-Myc expression was significantly upregulated at both the protein and mRNA levels following RBM38 knockdown in MCF-7 (Fig. 3c, d; *P* < 0.05) and ZR-75-1 (Fig. 3g, h; *P* < 0.05) cells. This indicated that RBM38 negatively regulates c-Myc expression in breast cancer cells.

### RBM38 destabilizes the c-Myc transcript by directly binding to c-Myc mRNA

**Fig. 4:**
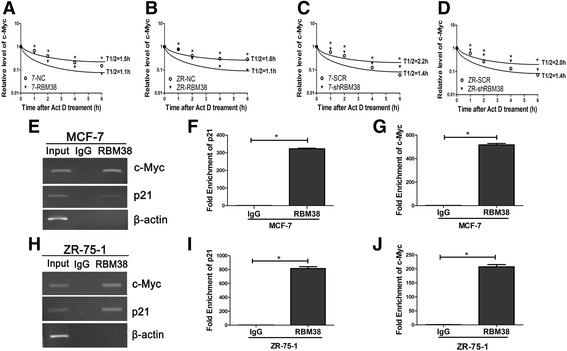
RBM38 destabilizes the c-Myc transcript by directly binding to c-Myc mRNA. **a**, **b** Ectopic expression of RBM38 shortened the half-life of c-Myc transcript. **a** MCF-7 (7) and **b** ZR-75-1 (ZR) cells were transfected with lentivirus to overexpress RBM38. The control (NC) and RBM38 overexpression (RBM38) cells were treated with 5 μg/ml actinomyclin D (Act D) for various times (0, 1, 2, 4, 6 h). Total RNAs were harvested, and then subjected to qRT-PCR analysis. **c**, **d** Knockdown of RBM38 lengthened the halflife of c-Myc transcript. **c** MCF-7 (7) and **d** ZR-75-1 (ZR) cells were transfected with the control (SCR) and RBM38 knockdown lentivirus (shRBM38). The following experiments were conducted as described in RBM38 overexpression. The relative quantification was calculated by the ΔΔCt method and normalized based on β-actin, *P < 0.05, then the logarithmic function analysis was used to assess the correlation. **e**–**j** RBM38 could bind to c-Myc transcript in vivo in breast cencer cells. **e**–**g** MCF-7 and **h**–**j** ZR-75-1 cell lysates were immunoprecipitated with RBM38 antibody or control IgG followed by RT-PCR (**e**, **h**) and qRT-PCR (**f**, **g**, **i**, **j**) measuring the levels of c-Myc, p21 transcripts in the RBM38 or IgG immunocomplexes. Upon normalization with the level of β-actin transcript, these data were calculated from three separate experiments and performed as mean ± SEM, *P < 0.05

The relative half-life of c-Myc mRNA decreased from 1.5 to 1.1 h following RBM38 overexpression in MCF-7 cells (Fig. 4a). Similarly, the relative half-life of c-Myc mRNA decreased from 1.6 to 1.1 h in ZR-75-1 cells (Fig. 4b). Furthermore, the relative half-life of c-Myc mRNA increased from 1.4 to 2.2 h following RBM38 knockdown in MCF-7 cells (Fig. 4c), and from 1.4 to 2.0 h in ZR-75-1 cells (Fig. 4d). These data suggested that RBM38 destabilizes the c-Myc transcripts. RIP assays were performed to evaluate the ability of RBM38 to bind to c-Myc transcripts in vivo. MCF-7 and ZR-75-1 cells were immunoprecipitated with RBM38 antibody or control IgG followed by RT-PCR (Fig. 4e, h) and qRT-PCR (Fig. 4f, g, i, j) analysis of the levels of c-Myc and p21 transcripts in the RBM38 or IgG immunocomplexes. The results showed that c-Myc mRNA was detectable in the RBM38 immunoprecipitates, but not in the control IgG immunoprecipitates in MCF-7 (Fig. 4e) and ZR-75-1 (Fig. 4h) cells. β-actin transcripts, which represented a negative control, were not bound by RBM38 (Fig. 4e, h). Conversely, the positive control p21 transcripts were also detectable in the RBM38 immunoprecipitates but not in the control IgG immunoprecipitates (Fig. 4e, h), which is consistent with previous reports [9, 10, 28]. This indicated that RBM38 destabilizes the c-Myc transcripts by binding directly to c-Myc mRNA.

### RBM38 binds directly to the AREs in the 3′-UTR of c-Myc mRNA

**Fig. 5:**
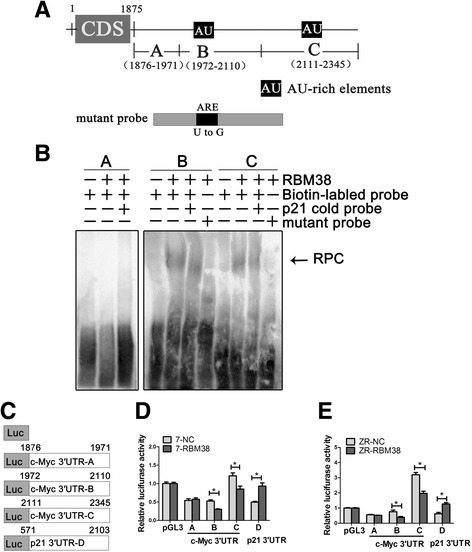
RBM38 directly binds to multiple regions in the 3′-UTR of c-Myc mRNA. **a** Schematic presentation of c-Myc transcript and the location of probes used for REMSA. AREs were shown in *shaded boxes*. The mutant probes were also stated briefly. **b** Probes B and C, but not probe A, were associated with RBM38. REMSA was performed by mixing probes A, B, C with RBM38 protein respectively. p21 cold probe, derived from 3′-UTR of p21 mRNA, was used as competitive probe. Competition assay was performed by the addition of an excess amount of p21 cold probe to the reaction mixture containing RBM38 protein and biotin-labeled probe. The *arrow* indicated RNA-protein complexes (RPCs). **c** Schematic representation of the luciferase plasmid with 3′-UTR-A, 3′-UTR-B 3′-UTR-C and 3′-UTR-D whose sequences were identical to probes A, B, C and p21 cold probe respectively. **d**, **e** The luciferase activity for the reporter carrying 3′-UTR-B or -C was repressed by RBM38. MCF-7 and ZR-75-1 cells with RBM38 overexpression lentivirus (RBM38) and the control (NC) were transfected with pGL3 control reporter or pGL3 reporter carrying 3′-UTR-A, 3′-UTR-B and 3′-UTR-C and 3′-UTR-D, separately. The fold changed in relative luciferase activity was a product of the luciferase activity induced by RBM38 divided by that induced by the NC. these data were calculated from three separate experiments and performed as mean ± SEM, *P < 0.05

To explore the RBM38-binding region (s) in vitro, the 3′-UTR of c-Myc mRNA was divided into three sections, sites A (probe A), B (probe B) and C (probe C) (Fig. **5**a). Among these, sites B and C were rich in AREs, as site A (probe A) contained no AREs (Fig. 5a). A probe designed based on the sequence of p21 mRNA 3′-UTR that has been shown to be bound by RBM38 was used as a competitive probe [9, 10, 28]. Following REMSA, RBM38 was mixed sequentially with probes A, B and C. Competition assays were performed by the addition of an excess amount of p21 cold probe to the reaction mixture containing RBM38 protein and biotin-labeled probes. The results showed that the RBM38 protein formed a complex (RPC) with probes B and C, but not with probe A (Fig. 5b). The formation of the complex was weakened by the addition of p21 cold probe and no RPC formation with RBM38 was observed following mutation of the AREs in probes B and C (mutant probes) (Fig. 5b). This indicated that RBM38 binds to the AREs in the 3′-UTR of c-Myc mRNA in vitro. To verify the requirement of these AREs for RBM38-mediated inhibition of c-Myc expression, dual-luciferase assays were performed using pGL3 reporters carrying 3′-UTR-A, 3′-UTR-B, 3′-UTR-C and 3′-UTR-D the sequences of which were identical to probes A, B, C and the p21 cold probe (Fig. 5c). The results indicated that the luciferase activity of a reporter carrying 3′-UTR-B and -C was greatly repressed by RBM38, while the 3′-UTR-A did not respond to RBM38 in MCF-7 (Fig. **5**d) and ZR-75-1 (Fig. 5e) cells. In addition, RBM38 increased the luciferase activity of a reporter carrying the 3′-UTR of the p21 mRNA (Fig. 5d, e), which is consistent with previous reports [9, 10, 28]. These data revealed that the AREs in the 3′-UTR of c-Myc mRNA were responsive to RBM38. Furthermore, both the 3′-UTR-B and 3′-UTR-C sites of c-Myc mRNA were sufficient for RBM38-mediated inhibition of c-Myc expression. This indicated that RBM38 binds to the AREs in the 3′-UTR-B and 3′-UTR-C sites of c-Myc mRNA to suppress c-Myc expression.

### Specific c-Myc inhibitors decrease RBM38-induced growth suppression in breast cancer cells

**Fig. 6:**
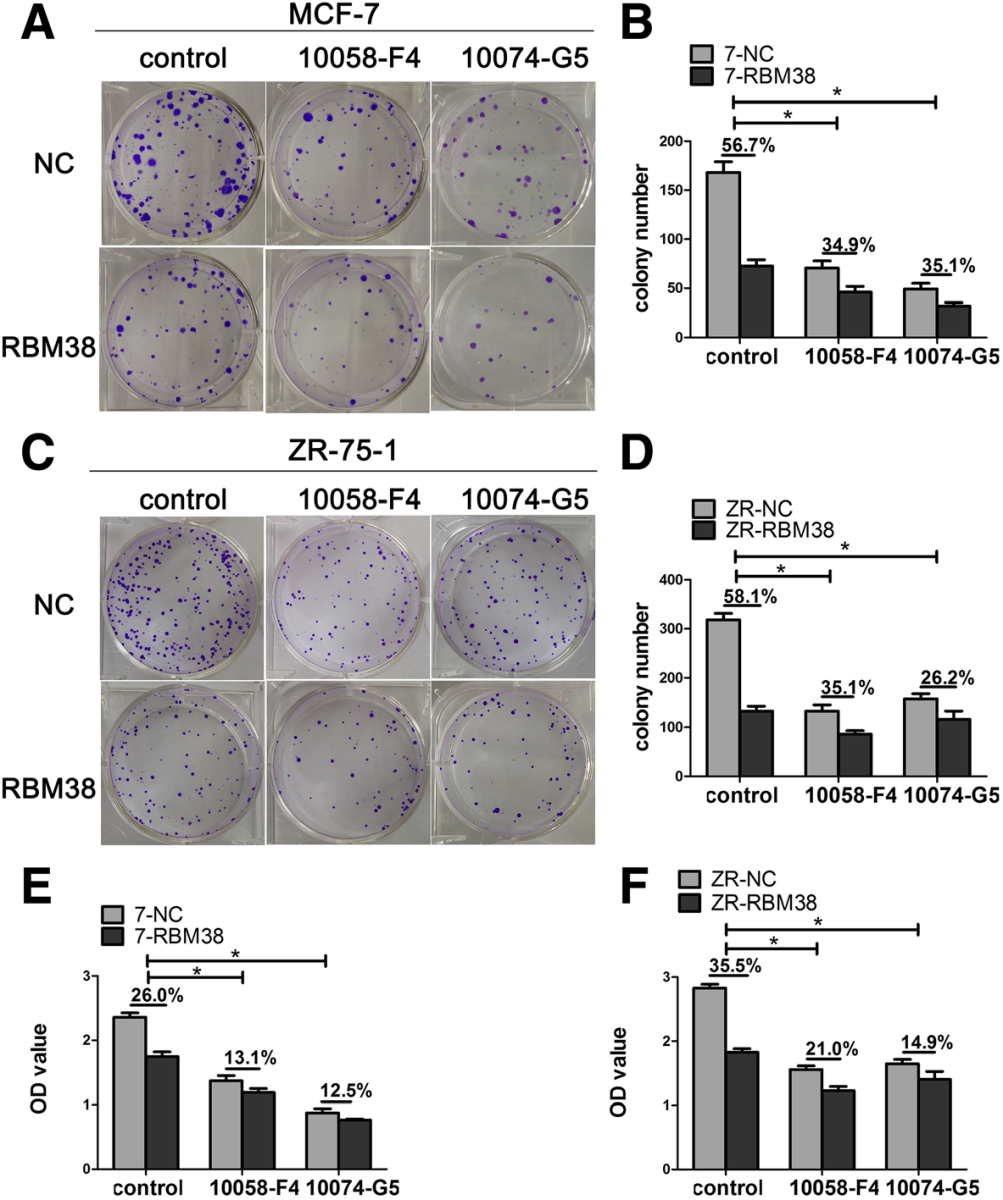
Specific c-Myc inhibitors decreases RBM38-induced growth suppression in breast cancer cells. **a**–**f** Colony formation assay and CCK-8 assay were performed to explore the growth suppression of RBM38 overexpression cells in the presence of specific c-Myc inhibitors. **a**–**d** MCF-7 and ZR-75-1 cells with RBM38 overexpression lentivirus (RBM38) and the control (NC) were treated with 15 nM 10058-F4, 15 nM 10074-G5 respectively. The growth of MCF-7 (**a**) and ZR-75-1 cells (**c**) over 21 d was measured using colony formation assays. Quantified data were obtained from colony formation assays in MCF-7 (**b**) and ZR-75-1 cells (**d**). The inhibition ratio of colony formation induced by overexpression of RBM38 was calculated by {colony number (NC)-colony number (RBM38)}/colony number (NC). The inhibition ratio of colony formation was significantly decreased by the addition of specific c-Myc inhibitors compared to control cells, respectively, *P < 0.05. **e** MCF-7 and **f** ZR-75-1 cells with RBM38 overexpression lentivirus (RBM38) and the control (NC) were treated with 70 nM 10058-F4, 70 nM 10074-G5 respectively over 3 d. The OD value was obtained from CCK-8 assays. The inhibition ratio of proliferation induced by overexpression of RBM38 was calculated by {OD value (NC)-OD value (RBM38)}/OD value (NC). The inhibition ratio of proliferation was significantly reduced in the presence of specific c-Myc inhibitors compared to control cells, *P < 0.05

To explore the potential of RBM38 to function as a tumor suppressor by repressing c-Myc expression in breast cancer, we used two specific c-Myc inhibitors, 10058-F4 and 10074-G5, which target c-Myc/Max dimerization and inhibit the c-Myc-Max interaction to prevent transactivation of c-Myc target genes [29, 30]. Ectopic expression of RBM38 suppressed proliferation of MCF-7 and ZR-75-1 cells, which is consistent with our previous finding [5]. In the colony formation assays, the inhibition ratio of colony formation induced by RBM38 overexpression was reduced from 56.7 to 34.9% or 35.1% following treatment of MCF-7 cells with either 10058-F4 or 10074-G5 for 21 d, respectively (Fig. 6a, b). Similarly, the inhibition ratio of colony formation decreased from 58.1 to 35.1% or 26.2% following treatment of ZR-75-1 cells with either 10058-F4 or 10074-G5, respectively (Fig. 6c, d). In CCK-8 assays, the inhibition ratio of proliferation induced by RBM38 overexpression was reduced from 26.0 to 13.1% or 12.5% following treatment of MCF-7 cells with either 10058-F4 or 10074-G5 for 3 d, respectively (Fig. 6e). Additionally, the inhibition ratio of proliferation was reduced from 35.5 to 21.0% or 14.9% following treatment of ZR-75-1 cells with either 10058-F4 or 10074-G5, respectively (Fig. 6f). This indicated that the inhibition of breast cancer cell proliferation induced by RBM38 is reduced by specific c-Myc inhibitors.

## Discussion

In this study, we demonstrated that c-Myc inhibited RBM38 expression by binding directly to the E-box motif in the promoter region of the RBM38 gene. In addition, RBM38 was found to destabilize c-Myc transcripts by direct targeting of AREs in the 3′-UTR of c-Myc mRNA, leading to suppression of c-Myc expression, and forming a novel RBM38-c-Myc loop involved in the regulation of breast cancer cell proliferation.

RBM38, which is a member of the RRM family of RBPs, mediates post-transcriptional regulation of the biological characteristics of target genes, such as members of the p53 family [11–13], p21 [9, 10, 28], the Hu antigen-R (HuR) [10, 31], mouse double minute 2 homolog (MDM2) [32], Cytokine-1 [33], E2F1 [8], PPM1D [34], estrogen receptors (ER) [25], and progesterone receptors (PR) [26]. RBM38 binds directly to AREs in the 3′-UTR of target genes to regulate their function by influencing the stability of the transcripts. Previously, RBM38 was found to be expressed at lower levels in breast cancer tissues compared to that in normal breast tissues, where it acts as a tumor suppressor [5]. In the present study, RBM38 overexpression decreased the levels of c-Myc protein and mRNA in breast cancer cells, whereas levels were increased by RBM38 knockdown. Moreover, RBM38 destabilized c-Myc transcripts by direct binding. In further studies, REMSA and dual-luciferase reporter assays were used to confirm that RBM38 binds directly to the two AREs in the 3′-UTR of c-Myc mRNA to inhibit its expression in breast cancer cells. The expression of c-Myc is known to be under tight control at both the transcription and translational levels. However, the regulation of c-Myc mRNA stability by RBPs is less well characterized [35]. In this study, we have identified a novel mechanism in which RBM38 destabilizes the c-Myc transcript by binding directly to target AREs in the 3′-UTR of c-Myc mRNA to suppress c-Myc protein expression.

To date, most studies have investigated the mechanism by which RBM38 regulates target genes at the post-transcriptional level, while RBM38 is regulated by other genes, such as p53 family members, and E2F1 [8, 9]. In this study, a novel post-transcriptional mechanism underlying the regulation of RBM38 expression was identified. In this study, RBM38 expression was significantly increased at both the mRNA and protein levels by c-Myc knockdown in breast cancer cells. Furthermore, ChIP studies showed that c-Myc binds directly to the presumed c-Myc binding site (E-box) in the RBM38 promotor region. Moreover, dual-luciferase reporter assays showed that the luciferase activity of a reporter carrying the RBM38 E-box greatly increased following c-Myc knockdown in the breast cancer cells. This indicates that c-Myc negatively regulates RBM38 expression in breast cancer cells by binding to the E-box motif in the promoter region of the RBM38 gene. Additionally, IHC analysis provided further evidence that RBM38 expression is negatively correlated with c-Myc expression in the breast cancer tissues.

c-Myc is upregulated in one-third of breast cancers, playing an important role in carcinogenesis and the survival prognosis of patients with breast cancer [36–39]. Moreover, c-Myc is a vital factor involved in anti-estrogen resistance, and has been identified as a radiosensitive locus in breast cancer [40, 41]. c-Myc now represents an interesting therapeutic target in breast cancer. In contrast, RBM38 is reported to inhibit tumor growth, exerting the opposite effect in breast cancer. To explore the role of the RBM38-mediated decrease in c-Myc expression in the RBM38-induced suppression of breast cancer, the specific c-Myc inhibitors, 10058-F4 and 10074-G5, were used to repress the transcriptional activity of c-Myc. Both specific inhibitors significantly decreased RBM38-induced growth suppression in breast cancer cells, indicating that the mechanism by which RBM38 acts as a tumor suppressor depends, at least partially, on its ability to repress c-Myc expression in breast cancer.

## Conclusions

RBM38 mediates direct inhibition of c-Myc expression, which in turn suppresses RBM38 expression. Thus, it can be concluded that RBM38 and c-Myc form a unique mutually antagonistic RBM38-c-Myc loop in breast cancer. This may provide new insight into the development of novel therapeutics for breast cancer.

## Additional files

Additional file 1: Table S1. Sequence of REMSA probes. **Table S2**. The primers used in quantitative RT-PCR. (DOC 38 kb)

Additional file 2: Figure S1. RBM38 and c-Myc cellular localization in breast cancer cells with immunofluorescence. IF staining of RBM38 in MCF-7 and ZR-75-1 cells at 400× magnification. Green represented c-Myc staining, Red represented RBM38 staining. Blue represented nuclear DNA staining with DAPI. Scale bars indicate 20 μm. (a, b) RBM38 and c-Myc were expressed in the cytoplasm and nucleus in breast cancer cells. (JPG 3735 kb)

## Abbreviations

3′-UTR: 3′-untranslated region
Act D: Actinomycin D
AREs: AU/U-rich elements
ChIP: Chromatin immunoprecipitation
ER: Estrogen receptors
HuR: Hu antigen-R
IF: Immunofluorescence
IHC: Immunohistochemical
PR: Progesterone receptor
RBP: RNA-binding protein
REMSA: RNA electrophoretic mobility shift assay
RIP: RNA immunoprecipitation
RRM: RNA recognition motif
