# Supplementary material for: The role of c-Myc-RBM38 loop in the growth suppression in breast cancer
Source: J Exp Clin Cancer Res. 2017 Apr 11;36:49. doi: 10.1186/s13046-017-0521-5 (PMC5387383; doi:10.1186/s13046-017-0521-5)
Supplement: Supplementary file 1 — Sequence of REMSA probes. Table S2. The primers used in quantitative RT-PCR. (DOC 38 kb) [file 13046_2017_521_MOESM1_ESM.doc]

**The role of c-Myc-RBM38 loop in** **the** **growth suppression in breast cancer**

Xiao-Xia Li^1,*^, Liang Shi^1,*^, Xu-Jie Zhou^1,*^, Jing Wu^1^, Tian-Song Xia^1^, Wen-Bin Zhou^1^, Xi Sun^1^, Lei Zhu^1^, Ji-Fu Wei^2^, Qiang Ding^1^.

^1^ Jiangsu Breast Disease Center, the First Affiliated Hospital with Nanjing Medical University, 300 Guangzhou Road, Nanjing 210029, China

^2^ Research Division of Clinical Pharmacology, the First Affiliated Hospital with Nanjing Medical University, 300 Guangzhou Road, Nanjing 210029, China

^*^ These authors contributed equally to this work

Correspondence to:

Qiang Ding, Jiangsu Breast Disease Center, the First Affiliated Hospital with Nanjing Medical University, 300 Guangzhou Road, Nanjing 210029, China;

e-mail: dingqiang@njmu.edu.cn.

Ji-Fu Wei, Research Division of Clinical Pharmacology, the First Affiliated Hospital with Nanjing Medical University, 300 Guangzhou Road, Nanjing 210029, China; email: weijifu@hotmail.com.

**Table S1: Sequence of** REMSA probes

| Probe | Sequence |
| --- | --- |
| A | TAATACGACTCACTATAGGGGGAAAAGTAAGGAAAACGATTCCTTCTAACAGAAATGTCCTGAGCAATCACCTATGAACTTGTTTCAAATGCATGATCAAATGCAACCTCACAACC |
| B | TAATACGACTCACTATAGGGTTGGCTGAGTCTTGAGACTGAAAGATTTAGCCATAATGTAAACTGCCTCAAATTGGACTTTGGGCATAAAAGAACTTTTTTATGCTTACCATCTTTTTTTTTTCTTTAACAGATTTGTATTTAAGAATTGTTTTTAAA |
| C | TAATACGACTCACTATAGGGAAATTTTAAGATTTACACAATGTTTCTCTGTAAATATTGCCATTAAATGTAAATAACTTTAATAAAACGTTTATAGCAGTTACACAGAATTTCAATCCTAGTATATAGTACCTAGTATTATAGGTACTATAAACCCTAATTTTTTTTATTTAAGTACATTTTGCTTTTTAAAGTTGATTTTTTTCTATTGTTTTTAGAAAAAATAAAATAACTGGCAAATATATCATTGAGCCAAA |
| P21  mRNA  3′-UTR | TAATACGACTCACTATAGGGTCTTAATTATTATTTGTGTTTTAATTTAAACACCTCCTCATGTACATACCCTGGCCGCCCCCTGCCCCCCAGCCTCTGGCATTAGAATTATTTAAACAAAAACTAGGCGGTTGAATGAGAGGTTCCTAAG |

**Table S2: The primers used in quantitative RT-PCR**

| β-actin | Forward | 5′-GCTGTGCTATCCCTGTACGC-3′ |
| --- | --- | --- |
| β-actin | Reverse | 5′-TGCCTCAGGGCAGCGGAACC-3′ |
| RBM38 | Forward | 5′-ACGCCTCGCTCAGGAAGTA-3′ |
| RBM38 | Reverse | 5′-GTCTTTGCAAGCCCTCTCAG-3′ |
| c-Myc | Forward | 5′-GGCTCCTGGCAAAAGGTCA-3′ |
| c-Myc | Reverse | 5′-CTGCGTAGTTGTGCTGATGT-3′ |
| p21 | Forward | 5′-TGTCCGTCAGAACCCATGC-3′ |
| p21 | Reverse | 5′-AAAGTCGAAGTTCCATCGCTC- 3′ |
