# Supplementary figures and images for: The role of c-Myc-RBM38 loop in the growth suppression in breast cancer
Source: J Exp Clin Cancer Res. 2017 Apr 11;36:49. doi: 10.1186/s13046-017-0521-5 (PMC5387383; doi:10.1186/s13046-017-0521-5)

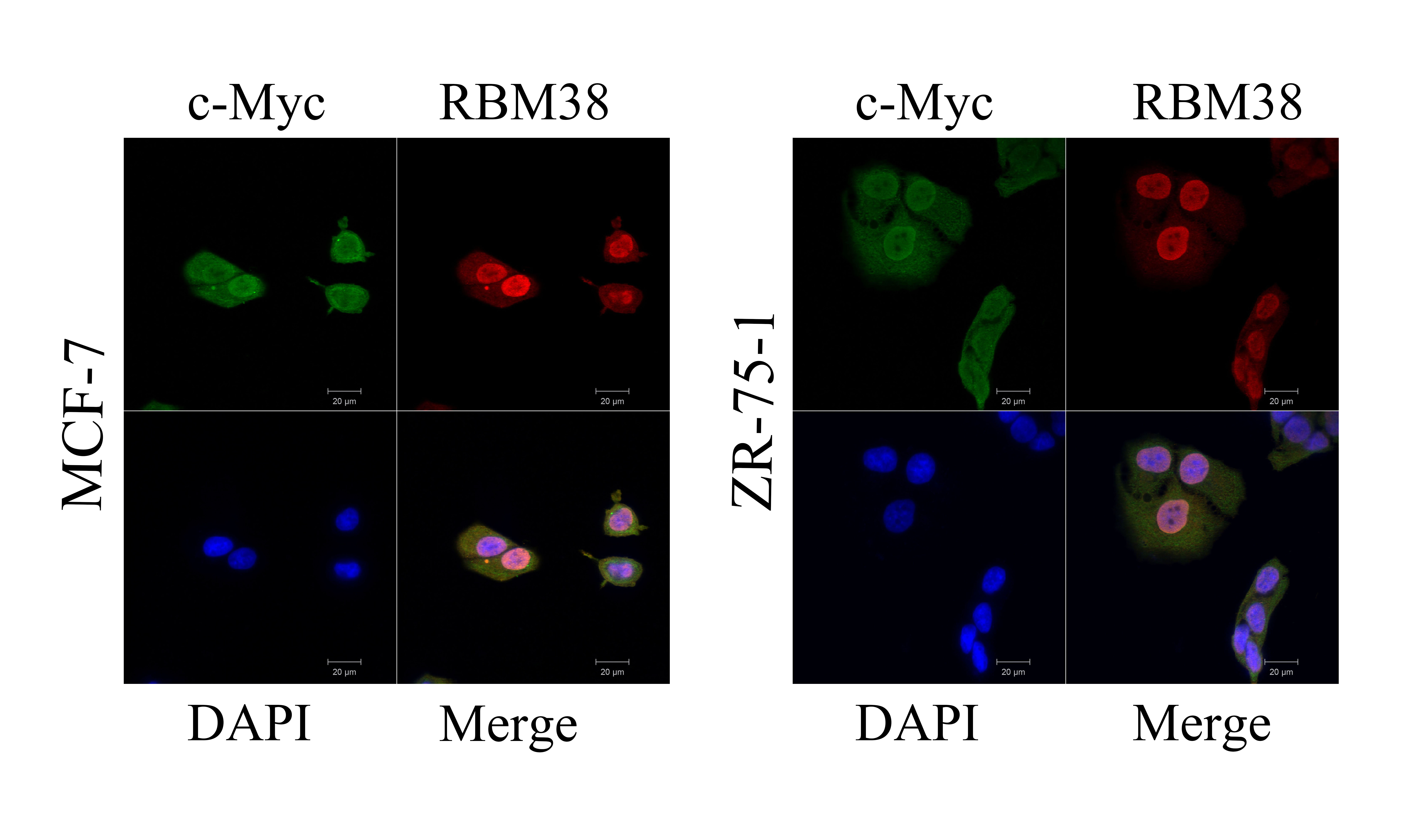

Supplement: Supplementary file 2 — RBM38 and c-Myc cellular localization in breast cancer cells with immunofluorescence. IF staining of RBM38 in MCF-7 and ZR-75-1 cells at 400× magnification. Green represented c-Myc staining, Red represented RBM38 staining. Blue represented nuclear DNA staining with DAPI. Scale bars indicate 20 μm. (a, b) RBM38 and c-Myc were expressed in the cytoplasm and nucleus in breast cancer cells. (JPG 3735 kb) [file 13046_2017_521_MOESM2_ESM.jpg]
